# Lipid profile in consecutive pregnancies

**DOI:** 10.1186/1476-511X-9-58

**Published:** 2010-06-05

**Authors:** David Mankuta, Matan Elami-Suzin, Asher Elhayani, Shlomo Vinker

**Affiliations:** 1 Hadassah University Hospital Jerusalem Israel; 2 Meir Medical Center Kefar Saba, Israel; 3 Department of Family Medicine, Sackler Faculty of Medicine, Tel Aviv University, Tel Aviv, Israel

## Abstract

**Aim:**

To describe the lipid profile of women prior to, during and after pregnancy and to assess the effect of consecutive pregnancies on the plasma lipid profile.

**Methods:**

Blood lipid levels of 1752 women aged 20-45 years who delivered between 1999 and 2005 were measured. The lipid profile included total cholesterol, LDL-C (Low density lipoprotein), HDL-C (High density lipoprotein-C), VLDL-C (Very low density lipoprotein) and triglycerides (TG). The measurements were classified into the following categories: non-pregnant state (12 months prior to conception), during the three trimesters of pregnancy and from 6 weeks to 12 months postpartum. This profile was tested in up to three subsequent pregnancies.

**Results:**

Total cholesterol levels overall rose during pregnancy. In the first trimester there is an average decrease of 11.4 mg/dL in total cholesterol level (p < 0.0001) followed by an average increase of 50.5 mg/dL and 28 mg/dL in the second and third trimesters respectively (p < 0.0001). In the year after pregnancy, the levels return to pre- pregnancy levels. LDL and triglyceride levels show a similar pattern.

In contrast, HDL-C levels do not change significantly in the first trimester. The second trimester is characterized by an average elevation of 14 mg/dL (p < 0.0001) and a decrease of 5 mg/dL in the third trimester (p = 0.03).

The average HDL-C levels of every period tested were lower in the 2nd and 3rd subsequent pregnancies.

**Conclusions:**

There is a general increase in total cholesterol, LDL and VLDL during pregnancy. We demonstrate a cumulative effect of consecutive pregnancies on lowering HDL cholesterol levels. This effect may have negative implications on future cardiovascular health.

## Introduction

Blood lipid concentrations, lipoproteins and apolipoproteins in the plasma increase significantly during pregnancy [1]. Fat storage occurs primarily during mid-pregnancy [2,3].There is some evidence that progesterone, which increases markedly in the second half of pregnancy, may act to reset the lipostat in the hypothalamus.

Hypercholesterolemia is an important cause of early atherosclerosis [4]. Nevertheless, there is conflicting evidence for an association between parity and the risk of cardiovascular disease in women [5,6]).

LDL-C levels peak at mid-third trimester, probably as a consequence of the hepatic effect of estradiol and progesterone [7]). It has been suggested that the increase in plasma triglycerides and LDL-C patterns during pregnancy might be used to identify women who will develop atherogenic changes later in life [1]).

Previous studies have reported a decline in HDL cholesterol up to 10 years after the first pregnancy, independent of weight, central adiposity and selected behavioral changes [8].

We undertook to evaluate the lipid profile prior to, during and post-pregnancy, in single and subsequent pregnancies, over a 6-year period, and to specifically examine the effect of up to three pregnancies on the lipid profile.

## Methods

The study was conducted in the Central District primary care clinics of Clalit Health Services. This is the largest health-management organization (HMO) in Israel, with approximately 3.5 million clients. The Clalit Health Services Registry database includes information collected from a Variety of sources: primary care physician reports, medication-use files, hospitalization records and outpatient clinic records.

The electronic medical records of 1752 women aged 20-45 women who delivered between January 1, 1999 and December 31, 2005 were reviewed. The women were all in the reproductive age, i.e. 20-45 years old. Adolescents and women over age 45 were excluded because pregnancy in those age groups is considered to be high risk. All the information on chronic diseases in the study population was provided by their primary care physicians based on CHPPC codes (Classification of Health Problems in Primary Care) for these diseases. Women with chronic diseases that may affect the lipid profile were excluded from the study. Furthermore, all the prescriptions that the study population used during the period were evaluated. Women who used a medication that could possibly affect the lipid profile were also excluded, as were patients with a family history of dyslipidemia.

The routine blood work for pregnant women in Israel and most other countries usually includes a complete blood count in the first trimester, and later syphilis and HbsAg screening; it does not as a rule include a lipid profile. Nevertheless, some physicians and patients elect to use the opportunity to perform additional tests. Although it is a minority of pregnant women in whom a lipid profile is evaluated, the absolute numbers are high. It is possible that the group requesting or being offered the test may be more concerned or aware of blood lipid status. The methods of registry acquisition and maintenance have been described in detail elsewhere [9].The lipid profiles obtained were classified into the following categories: non-pregnant state (12 months prior to conception), during each trimester of pregnancy and from 6 weeks to 12 months post-partum. All lipid profiles of the same woman performed during the study period were retrieved. In general, the tests were performed following an overnight fast, although we did not have information on individual compliance with this requirement. The number of tests per patient ranged from one to six tests. The average results were analyzed with respect to each pregnancy for the whole group. Patients who were being treated medically for hypertension, diabetes or hypercholesterolemia (total cholesterol > 200 mg/dL) were excluded from the study.

### Statistical analysis

Tests and data preparations were analyzed using SAS ver. 8.1 Statistical package (SAS Institute Inc., Cary, NC, USA) with double sided 0.05 significance level. An independent two-sample t-test was used. If the distribution was too asymmetric we used the two-sample Mann-Whitney test.

Since the analyses were performed retrospectively on an existing database, and some women had lipid tests at different time intervals whereas others were tested on a single occasion, we performed separate analyses in those groups (paired analyses and two samples independent test, respectively). Any group sample size below 30 was excluded from analysis.

## Results

2686 test results were analyzed. Seventy-four women (4.3%) had one pregnancy during the six-year period of the study, 1567 (91.1%) had two pregnancies, and 79 (4.6%) had three pregnancies. The mean age of women at delivery was 30.4 years.

### Total cholesterol

**Figure 1:**
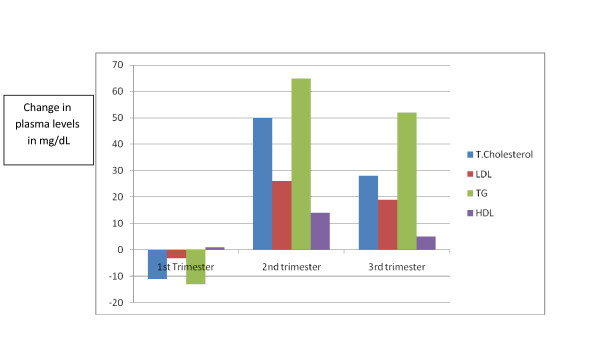
**Average lipid profile during pregnancy**.

Figure 1 depicts the various lipids and lipoprotein levels during pregnancy. Total cholesterol changes during pregnancy: a decrease of 11.4 mg/dL in total cholesterol level (p < 0.0001) is seen in the first trimester during the study period. A significant average increase of 50.5 mg/dL (p < 0.0001) occurs between the first and second trimesters, and a further increase of 28.5 mg/dL, on average, between the second and third trimesters (p < 0.0001). In the year after the pregnancy, the levels return to pre-pregnancy levels. A similar pattern of total cholesterol levels recurs in the second and third pregnancies.

### LDL cholesterol

The LDL-C levels decreased non-significantly by 3.3 mg/dL on average (p = 0.216) during the first trimester. During the second trimester LDL-C increases by 25.9 mg/dL (p < 0.0001), followed by a further increase of 19.4 mg/dL (p = 0.0097) in the third trimester. Within one year post-partum, LDL-C returns to pre-pregnant levels and continues to decline to levels even lower than pre-pregnancy during the second and third years post-partum.

### Triglycerides

The first trimester shows an average decrease of 13.3 mg/dL (p < 0.0001) in TG levels. The second trimester demonstrates an average elevation of 64.9 mg/dL (p < 0.0001) followed by a further increase of 52.2 mg/dL (p < 0.0001, paired t-test) in the third trimester.

### HDL cholesterol

**Figure 2:**
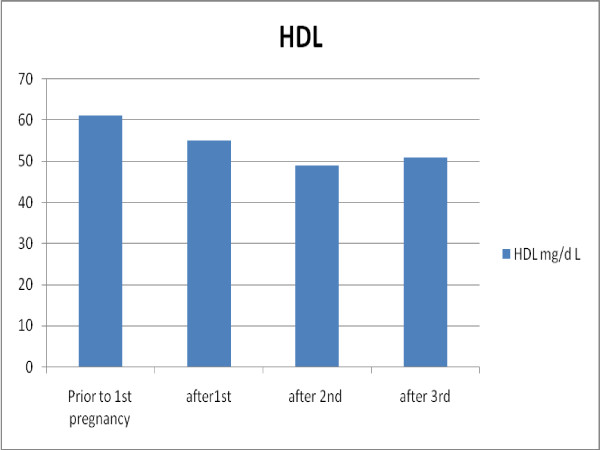
**Average HDL cholesterol levels in subsequent pregnancies**.

In contrast to total and LDL cholesterol, HDL-C levels do not change significantly in the first trimester. The second trimester is characterized by a steep elevation of 14 mg/dL in HDL-C levels (p < 0.0001) and a slight decrease of 5 mg/dL in the third trimester (p = 0.03). The levels plateau and do not decline further one year after pregnancy. Average HDL-C levels of every period tested were lower in subsequent pregnancies (Figure 2).

Table 1 summarizes the effects of pregnancies and post partum period on lipid/lipoprotein levels.

**Table 1:** Summary lipid profile during pregnancy

	One year after 3 subsequent pregnancies	3^rd ^trimester	2^nd ^trimester	1^st ^trimester
Total cholesterol	↑	↑↑↑	↑↑	═

LDL cholesterol	═	↑ ↑	↑	↓

HDL cholesterol	═	↑ ↑ ↑	↑	↓

Triglycerides	↓	↑ ↑ ↑	↑ ↑	N/A

## Discussion

Pregnancy induces significant metabolic changes. The concentrations of lipids, lipoproteins and apolipoproteins in the plasma increase appreciably during pregnancy. The lipid levels are affected by maternal hormonal changes (rise in insulin, progesterone, 17-β estradiol and Human Placental Lactogen). Other maternal factors such as BMI (body mass index), maternal weight gain, maternal nutrition, pre-pregnancy lipid levels and various medical complications of pregnancy may also have significant effects on lipid metabolism and plasma levels [10].

Until the CARDIA study by Gunderson et al [11,12] evidence regarding the association between parity and the risk of cardiovascular disease in women has been conflicting. That prospective study, among 1952 American women, examined lipid changes over a 10 year period, and adjusted the results to allow for various confounding factors. For both white and non-white populations a decline of 3 to 4 mg/dL in HDL-C was seen during the first pregnancy compared with nulliparous women. Higher order births were not associated with a greater decline in HDL cholesterol.

We assessed the influence of consecutive pregnancies on lipid changes. In our study population the total cholesterol generally increased during pregnancy by approximately 40% and returned to pre-pregnancy levels within one year postpartum. We noted a slight decrease during the first trimester, an observation described in other studies [13,14].A possible explanation for the decrement during the first trimester may be a decreased intake of food due to nausea and vomiting, which characterize the early stages of pregnancy (10).

LDL-C demonstrates a similar pattern to total cholesterol, with an average increase of approximately 23% in the third trimester. However, after the first year postpartum, the levels of LDL-C continue to decline even below pre-pregnancy levels.

Triglyceride levels, as also shown in other studies [1,14], doubled in the third trimester. However, we also demonstrated an initial decrease in TG levels in the first trimester, a pattern not shown in previous studies [1,14]. Apparently, consecutive pregnancies do not influence baseline (non-pregnant) TG levels. These findings are consistent with the findings of Gunderson et al [11,12].

HDL-C levels mainly rise in the second trimester, but begin to decline from the third trimester, reaching their nadir one year postpartum. Interestingly, our results show that with each consecutive pregnancy HDL-C rises to lesser extent, and the nadir is lower. These changes may be a risk factor for future atherosclerosis.

This study has several limitations. The first is that it lacks a non-pregnant control group in the same period. It is known that aging is a significant factor affecting changes in the lipid profile - a factor we did not analyze. We also lack data about weight changes during this six-year period, as well as information on the diet of the subjects - factors that may affect the lipid profile [15]). Another possible explanation for the changes we found may be the decline in physical activity due to increased demands of the growing family over time.

Our study has some other limitations. This is a retrospective study and the effect of factors such as breast feeding and birth control pills were not available for adjustment [10-12].We controlled the women to their own pre-pregnancy lipid levels rather than comparing to a nulliparous group, which would provide another perspective of the change in the lipid profile with age.

Our study reinforces the results of previous studies [14,15] supporting the hypothesis that pregnancy exerts persistent adverse effects on HDL cholesterol. We demonstrate a cumulative effect of consecutive pregnancies on lowering HDL cholesterol levels. This effect may have negative implications on future cardiovascular health.

## Competing interests

The authors declare that they have no competing interests.

## Authors' contributions

DM initiated the study idea, analyzed the data, wrote the manuscript formed the graphs and table and submitted the manuscript and is the corresponding author, ME has contributed to the statistical analysis of the study, and the writing of the manuscript and has equal contribution to the manuscript as the first author. AE contributed in the database establishment and data retrieval while SV came with the idea of the study with DM and performed the data retrieval and analysis. All authors have read and approved the final manuscript
